# Results of Double Blind Placebo Controlled Trial to Assess the Effect of Vitamin B6 on Managing of Nausea and Vomiting In Pediatrics with Acute Gastroenteritis

**DOI:** 10.5539/gjhs.v5n6p197

**Published:** 2013-09-29

**Authors:** Hojjat Derakhshanfar, Arash Hadian Amree, Hossein Alimohammadi, Majid Shojahe, Ali Sharami

**Affiliations:** 1 Pediatric Emergency Medicine Department, Mofid Children Hospital, Shahid Beheshti University of Medical Sciences, Tehran, Iran; 2 Deputy of Education, Mazandaran University of Medical Sciences, Sari, Iran; 3 Emergency Medicine Department, Shahid Beheshti University of Medical Sciences, Tehran, Iran

**Keywords:** gastroenteritis, vomiting, dehydration, vitamin B6

## Abstract

**Background:**

Gastroenteritis and respiratory tract infections are the most common childhood diseases. Despite the common use of vitamin B6 to control vomiting in children with gastroenteritis, no study has been performed in this field. This study aimed to assess the value of vitamin B6 in the prevention of vomiting in patients with mild to moderate gastroenteritis.

**Methodology:**

This study was a double blind controlled clinical trial on 96 children with mild to moderate gastroenteritis with age range of 6 months to 12 years admitted in Mofid Children’s Hospital, Shahid Beheshti University of Medical Sciences, Tehran, Iran. Patients were randomly assigned to two groups of 48 subjects matched for age, sex and symptoms of dehydration. Vitamin B6 was given in case group and control group was given placebo. The severity of dehydration and vomiting in patients before and after treatment were evaluated by a physician. All data were recorded in the questionnaire and results in the two treatment groups were compared by using SPSS software (Version 15, Chicago, IL, USA).

**Results:**

The mean ± SD age of patients whom underwent Vitamin B6 treatment was 2.9±2.4 versus 2.5±2 in placebo group. Significant difference between mean age, gender, and severity of dehydration in children of two groups wasn’t observed. After treatment in both treatment groups, 40 patients (83.3%) had mild dehydration, and 8 patients (16.7%) had moderate dehydration. Vomiting was noted in 28 patients (58.3%) after treatment with vitamin B6 and in 37 patients (77.1%) after treatment with placebo. The mean frequency of vomiting after treatment with vitamin B6 was 1.7±1.3 times and in the control group (treated with distilled water) was 1.5±0.77 time, but no significant difference between the severity of dehydration, controlling vomiting and the mean frequency of vomiting was observed in both groups (P>0.05).

**Conclusion:**

It seems that the use of oral vitamin B6 treatment has no benefit and impact compared with the placebo. Thus, use of vitamin B6 in the prevention of vomiting due to acute mild to moderate gastroenteritis is not only scientifically, but in the present study it was proved to be ineffective. This work was done on a comparative basis and further researches are recommended.

## 1. Introduction

Acute gastroenteritis is the third reason of mortality worldwide (Navaneethan et al, 2008). Bacteria, parasites and viruses are the main predisposal agents. The correlated viruses includes rotaviruses, calciviruses and sapoviruses, enteric adenoviruses, human astroviruses, Aichiviruses, toroviruses, coronaviruses and picobirnaviruses and Enteroviruses (Wilhelmi et al., 2003; Phan et al., 2005). One of the major the presenting symptoms of gastroenteritis involve nausea and vomiting (Chitambar et al., 2012).

Nausea and vomiting decrease the quality of life. Patients sense a loss of appetite and discomfort. Following two weeks after nausea and vomiting, patients may be pessimistic and chance for suicide will increase (Qi You et al., 2009).

Nausea and vomiting is common finding in cancer patients. The American Society of Clinical Oncology suggests potential 5-HT3 receptor antagonists beside corticosteroids before chemotherapy to subjects receiving chemotherapy that are at high risk of emesis (Maranzano et al., 2005).

Although there is a lot of modern antiemetic agents, but still some patients are discomfort with current agents and the need for measurement of alternative treatments is required.

Vitamin B6 is considered as the first-line treatment for patients suffering nausea and vomiting during or after chemotherapy (Mazzotta et al., 2008). In a multiple-day, multiple-drug combination chemotherapy design, emetic responses are worsened by the different agents (Dario et al., 2008).

Vitamin B6 can relieve the symptoms which are caused by nausea and vomiting, including anorexia, indigestion, weak, anxiety, and hands and feet numbness (Fugh-Berman et al., 2003). In this regard, studies reported vitamin B6 can be used for the management of nausea and vomiting during pregnancy (Madjunkova et al., 2013; Dror et al., 2012; Aikins, 1998).

But still there is lack of information about the use of vitamin B6 on nausea and vomiting during gastroenteritis. Therefore, this study was conducted to evaluate the potential role of Vitamin B6 on nausea and vomiting in pediatrics.

## 2. Methods and Patients

This study was a double blind randomized placebo -controlled clinical trial on 96 children with mild to moderate gastroenteritis with age range of 6 months to 12 years whom were admitted in Mofid Children’s Hospital, Tehran, Iran (registration number: 89-01-119-7420).

Patients were randomly assigned to two groups of 48 subjects matched for age, sex and symptoms of dehydration. Vitamin B6 was given in case group and control group was given placebo (double blinded including the patients and researchers). The severity of dehydration and vomiting in patients before and after (recording the number of vomiting episodes in the 6 hour) treatment were evaluated by a physician.

Inclusion criteria included patients with age of 6 month to 12 years old, mild to moderate gastroenteritis and if there was no indications for intravascular injection.

Exclusion criteria involved patients out of defined range for age, severe gastroenteritis, history of antiemetic agents 10 days before evaluation and if the patients or their family was unsatisfied with cooperation.

### 2.1 Ethics

All subjects gave their consent to participate in the study. This study was conducted in accordance with the Declaration of Helsinki and good clinical practice according to International Conference on Harmonisation guidelines.

### 2.2 Statistical Analysis

Values were presented as Mean ±Standard deviation (SD). For statistical analysis, SPSS software (Version 15, Chicago, IL, USA) was used applying chi square and T test. *P* < 0.05 was defined as significant.

## 3. Results

The mean ± SD age of patients whom underwent vitamin B6 treatment was 2.9± 2.4 versus 2.5 ±2 in placebo group. In this relation, there was no significant change between the age of study arms (p=0.31).

28 (58.3%) patients in vitamin B6 group and 30 (62.5%) subjects in placebo group were male while 20 (41.6%) in vitamin B6 group and 18 (37.5%) in placebo group were female. There was no significant change in studied patients gender (p=083, CI 95%=0.5-2.7, Odds ratio=1.19).

Mild dehydration was observed in 41patients (85.4%) of Vitamin B6 group and in 40(83.3%) patients of placebo group. There was no statistically difference between the primary dehydration of Vitamin B6 and placebo group (p=0.1, CI 95%=0.28-2.57, Odds ratio=0.85) (Table 1).

**Table 1:** Profiles of primary dehydration in study populations

Patients	Primary Dehydration (Mild)	Primary Dehydration (Moderate)	Total
Vitamin B6	41(85.4%)	7(14.6%)	48(50%)
Placebo	40(83.3%)	8(16.7%)	48(50%)
Total	81(84.4%)	15(15.6%)	96(100%)

Mild dehydration after treatment was observed in 40 (83.3%) of Vitamin B6 group and in 40(83.3%) patients of placebo group. In this regard, there was no significant correlation between severity of dehydration after treatment (p=1, CI 95%=0.34-2.93, Odds ratio=1) (Table 2).

**Table 2:** Characteristic of dehydration after treatment after vitamin B6 and placebo

Patients	Dehydration (Mild)	Dehydration (Moderate)	Total
Vitamin B6	40(83.3%)	8(16.7%)	48(50%)
Placebo	40(83.3%)	8(16.7%)	48(50%)
Total	80(83.3%)	16(16.7%)	96(100%)

Vomiting was managed in 28 (58.3%) of Vitamin B6 group and in 37(77.1%) patients of placebo group. There was no significant change in treatment of vomiting between two study arms (p=0.08, CI 95%=0.99-5.82, Odds ratio=2.4).

**Figure 1:**
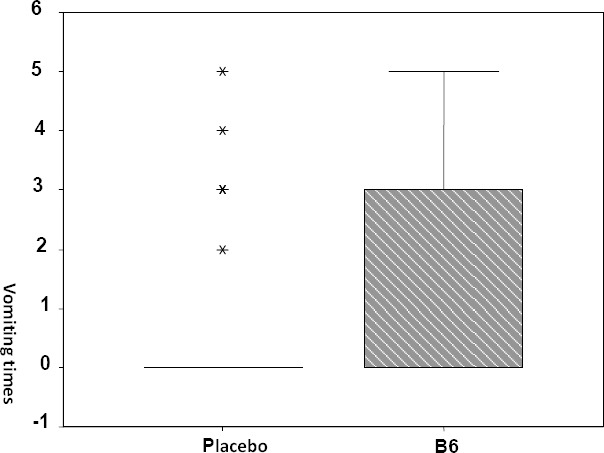
Times of vomiting after treatment between Vitamin B6 group and placebo group

The mean times of vomiting in Vitamin B6 group were 1.3±1.7 whereas the mean times of vomiting in placebo group were 1.5±0.77 with range of 0 to 5 times. There was no significant difference in times of vomiting between two groups (p=0.1) (Figure 1).

## 4. Discussion

In this study, we examined the effect of vitamin B6 versus placebo in management of vomiting in pediatrics acute gastroenteritis. Our study revealed that the prescription of vitamin B6 does not have curable impact on vomiting in children.

Vitamin B6 is a water-soluble vitamin which is important coenzyme in the metabolism of amino acids, carbohydrates, and lipids (Wibowo et al., 2012). Most researchers believe that the serum level of vitamin B6 is associated with the severity of nausea and vomiting (Ashkenazi-Hoffnung et al., 2013).

There are some theories behind this phenomenon. One theory is that vitamin B6 may have a coenzyme role in lysine reactivity (lysine being a protein residue of steroid hormone receptors), and lysine reactivity would rise the estrogen levels in pregnant women and the result is decrease in the severity of nausea and vomiting. On the other hand it seems that Vitamin B6 have a potential role in the synthesis of serotonin, dopamine, norepinephrine, and gamma–amino butyric acid by catalyzing the decarboxylation process.

Any disturbance in the latter neurotransmitter might cause nausea and vomiting during pregnancy. Therefore, the serum concentration of vitamin B6 in pregnant women may be correlated with nausea and vomiting (Miller, 1999; Sahakian et al., 1991).

The vomiting reflex is a complicated process organized by the vomiting center in the brainstem that receives stimuli from the periphery via afferent neurons of the vagus nerves in the autonomic nervous system or centrally via the chemoreceptor trigger zone, area postrema, or nucleus of the solitary tract. Many transmitters like dopamine, histamine, muscarine, and serotonin, are included in this phenomenon (Watcha et al., 1992; Kovac, 2000; Gan et al., 2003; Conway, 2009).

Wibowo et al. (2012) examined whether supplementation with vitamin B6 has any effect on nausea and/or vomiting in pregnancy. They showed that women with nausea and/or vomiting in pregnancy had significantly lower concentration of serum vitamin B6 in comparison to those without this symptom. Also they revealed that Vitamin B6 supplementation increased the plasma level of vitamin B6 concentration. They concluded that vitamin B6 supplementation is unlikely to impose the severity of symptoms. In another study, Qi You et al (2009) evaluated the vitamin B6 points PC6 injection during acupuncture on nausea and vomiting in patients with ovarian cancer. They reported that that acupuncture plus vitamin b6 pc6 point injection is a competent procedure against emesis in cancer patients undergoing chemotherapy.

Ben-Aroya et al. (1998) reported that pregnant women with mild nausea and/or vomiting had a significantly lower BMI in comparison with women with severe symptoms (21.8±3.5 vs 24.4±4.7; P≤0.05). There were no significant difference in the serum levels of protein and amino acids including tryptophan, could be changed to niacin and serotonin by the coenzyme activity of vitamin B6, between two study arms. They revealed that both studied groups had similar plasma protein levels but various concentration levels of vitamin B6, and that these alterations in serum levels affect the symptoms.

## 5. Conclusion

Besides these controversial reports about the effect of vitamin B6 and its supplementation on vomiting, our current study indicated that there is no useful effect in treatment of pediatric nausea and vomiting following acute gastroenteritis. Although our study evaluated the acute role of vitamin B6 on nausea and vomiting, there is need for more studies to examine the long effect of vitamin B6.
